# Ozone water or chloroxylenol: The comparison of disinfection effectiveness against the number of bacterial colonies in dental extraction instruments at the Universitas Sumatera Utara Dental and Oral Hospital

**DOI:** 10.12688/f1000research.132941.4

**Published:** 2025-07-31

**Authors:** Hendry Rusdy, Rahmi Syaflida Dalimunthe, Ahyar Riza, Ismahani Akilah D

**Affiliations:** 1 Universitas Sumatera Utara, Medan, North Sumatra, Indonesia

**Keywords:** Ozone water, Chloroxylenol, Bacterial Colony

## Abstract

**Background:**

The application of disinfectants on dental instruments is one way to prevent cross-infection. The urgency in this research is ozone water as a disinfectant is still limited and no research has been found on the use of ozone water on tooth extraction instruments. However, ozone has antibacterial properties so researchers are interested in studying the effectiveness of ozone water as a disinfectant when compared to the commonly used 4.8% chloroxylenol. This study aimed to determine the effectiveness of disinfection of ozone water and 4.8% chloroxylenol in reducing the number of bacterial colonies on dental extraction instruments at the Universitas Sumatera Utara Dental and Oral Hospital.

**Methods:**

The samples used were mandibular molar forceps have been used in tooth extraction procedures. This study was experimental and used 30 sample, where each group consisted of 10 tooth extraction instruments. The treatment group used ozone water and 4.8% chloroxylenol and the negative control group was cleaned with distilled water. The ozone water group was produced with an ozone generator with a concentration of 15 mg/L for 20 minutes. Then the active side of tooth extraction forceps was soaked for 30 minutes. In the 4.8% chloroxylenol (dettol®) group, the active side of the mandibular molar tooth extraction forceps was soaked in 250 mL for 60 minutes. In the control group, the mandibular molar tooth extraction forceps were rinsed with distilled water. After that, the sample was counted using a bactery colony counter.

**Results:**

The results of the data analysis showed a p-value ≤0.001, which means there was a significant difference in the disinfection using ozone water and 4.8% chloroxylenol on the number of bacterial colonies on dental extraction instruments.

**Conclusions:**

From this study it can be said that ozone water is more effective in disinfecting than 4.8% chloroxylenol.

## Introduction

Dental health workers are a group that is vulnerable to infection. ^1^ In dental practice, microorganisms can spread through blood, saliva or droplets through direct or indirect contact. One route of cross-infection due to indirect contact is tooth extraction instruments that have been contaminated with pathogenic microorganisms. ^2^ Tooth extraction procedures have a high risk of infection transmission due to contact of the instruments with the patient’s blood and saliva. ^3^ These must be aseptic at the time of tooth extraction to avoid bacterial infection, which is one of the complications that can occur as a result of tooth extraction. ^4^ If the tooth extraction instrument is contaminated, microorganisms can be transmitted to dentists, nurses, or other patients who will be infected with diseases. ^4^ According to the American Dental Association (ADA), it is estimated that dentists and patients may be exposed to around 40 types of infectious diseases when carrying out dental treatment procedures. Therefore, it is necessary to exercise prevention by disinfecting dental extraction instruments. ^2^

Alternative materials for disinfection in dentistry have been developed. Ozone water can function as a disinfectant with the ability to oxidize amino acids and destroy proteins in the cellular membranes of microorganisms. ^5^ Ozone water has the ability to kill pathogenic microorganisms and the resulting wastewater is safe to enter waterways. ^6^ Chloroxylenol or para-chloro-meta-xylenol (PCMX) is a chemical-level disinfectant commonly used to disinfect skin and surgical instruments in dentistry. ^7^ 4.8% chloroxylenol has a broad-spectrum antibacterial ability that can kill most bacteria and fungi. 4.8% chloroxylenol works by denaturing proteins, changing the permeability of cell walls and causing cell leakage. 4.8% chloroxylenol is one of the most widely used disinfectants in dentistry. 4.8% chloroxylenol is one of the phenol groups, and works by denaturing proteins, changing the permeability of cell walls and causing cell leakage. ^8^

Research conducted by Ochie K and Ohagwu C in 2009 demonstrated that 3.5% sodium hypochlorite was the most effective in disinfecting x-ray equipment and accessories, followed by methylated spirit, 4.8% chlorocylenol, and 2% dichlorocylenol. ^9^ A study by Igizeneza *et al.* in 2020 revealed that 4.8% chlorocylenol was highly efficient in eradicating *Staphylococcus* sp. and *Streptococcus* sp. strains by 100%. Guther's research in 2020, focusing on skin surfaces and hospital environments, discovered that 4.8% chlorocylenol could inhibit the metabolic activity of 38.9% of gram-positive bacteria and 60.7% of gram-negative bacteria.

Research on the effectiveness of ozone water (10 mg/L) as a disinfection agent on diamond burs that have been contaminated by *Streptococcus aureus, Escherichia coli, Candida albicans* and spores of *Bacillus atrophaeus* showed a reduction of microorganisms by 90.15-99.33%. ^9^ Research on the effectiveness of 4.8% chloroxylenol as a disinfecting agent showed its ability to interfere with the metabolic activity of Gram-positive bacteria at 38.9% concentration and Gram-negative at 60.7% concentration. ^10^

There are many disinfection agents that can be used in dentistry. Hypochlorous acid (HOCl) was evaluated as an antimicrobial disinfectant for alginate impression materials using a spraying technique. Research conducted by Bayan *et al.* HOCl at a concentration of 200 ppm for 5 and 10 minutes was compared with a negative control (no treatment) and a positive control using 0.5% sodium hypochlorite. Antimicrobial activity against Candida albicans, Staphylococcus aureus, and Pseudomonas aeruginosa was assessed using colony forming unit tests, alongside tests for dimensional stability and reproduction of details. Results indicated that HOCl exhibited significant antimicrobial activity against all tested microorganisms. Importantly, HOCl did not affect the dimensional stability of the alginate impression material. Therefore, HOCl has the potential to be an effective antimicrobial agent for alginate impression materials without compromising their surface details or dimensional stability. ^11^

Sodium hypochlorite is one of the original and most widely used disinfectants, effective against a broad spectrum of microorganisms including Human Immunodeficiency Virus, viruses, fungi, bacterial species, and their spores. A study conducted by Aseel M. Al-khafaji, Shorouq M. Abass, and Bayan S. Khalaf explored the use of SOLO and sodium hypochlorite disinfectant solutions for the disinfection of dental casts by spraying the stone specimen. This research aimed to assess the surface hardness, dimensional accuracy, reproduction of details, and surface porosity of type III, type IV, and type IV extra hard dental stone. The findings revealed no significant differences in dimensional accuracy, reproduction of details, surface hardness, and surface porosity among all types of tested stone samples after immersion or spraying with SOLO and sodium hypochlorite, except for type IV extra hard stone, which showed significant differences in surface hardness after spraying with SOLO and in surface porosity after immersion in both SOLO and sodium hypochlorite solutions. ^12^

Improper cleaning and maintenance of dentures can lead to contamination by various microbial pathogens, contributing to oral health issues. In a study by Maha A. Mahmood, Bayan S. Khalaf, and Shorouq M. Abass, HOCl was investigated to compare its antimicrobial effectiveness with oxalic, tartaric, citric acids, and alkaline peroxide, along with microwave irradiation, on the growth of Candida albicans and Staphylococcus aureus, respectively. Microwave irradiation was used to disinfect specimens of heat-activated acrylic resin and soft liner material. Microwave treatment or tartaric acid achieved sterilization of both heat-cured acrylic resin and soft lining material contaminated with Staphylococcus aureus, while microwave energy, oxalic acid, tartaric acid, and alkaline peroxide achieved complete sterilization against Candida albicans. Microwave irradiation was found to be the most effective method for sterilizing both heat-cured acrylic resin and soft lining material contaminated with either Staphylococcus aureus or Candida albicans, with tartaric acid ranking second. ^13^

This study aimed to compare the effectiveness of ozone water and 4.8% chloroxylenol disinfectants on tooth extraction instruments, because these critical instruments have a high risk of causing infection as they penetrate the mucous membranes of the oral cavity. ^9^

## Methods

### Study type

This research was a laboratory experimental research with post-test-only control group designs. The treatment group used ozone water and 4.8% chloroxylenol (dettol ^®^) and the negative control group was cleaned with distilled water (aquadest).

### Location

Universitas Sumatera Utara Dental and Oral Hospital, Laboratory of Microbiology, Faculty of Pharmacy, Mathematics and Natural Science University of Sumatera Utara, Medan, Indonesia.

### Duration

The study duration was about 3 months, from October to December.

### Sampling

The sampling technique used in this study was purposive sampling with 30 mandibular molar tooth extraction forceps as the sample. There were three treatment groups: the ozone water treatment group with 15 mg/L concentration, the 4.8% chloroxylenol treatment group, and the negative control (Aquadest) with 10 molar tooth extraction forceps in each treatment group. ^7^

### Ozone water production

Ozone water was prepared using an ozone generator with an ozone water concentration of 15 mg/L. The sterile nierbeken container is filled with 250 mL of distilled water, the container is inserted with a hose from the ozone generator. The timer on the ozone generator machine is set for 20 minutes to change the mineral water into ozone water. ^14^

### Disinfection procedure

In the ozone water and 4.8% chloroxylenol treatment group, the mandibular molar tooth extraction forceps were rinsed under running water, brushed with an antiseptic solution and rinsed again until the forceps were free of blood and saliva before soaking in a disinfecting solution. After that, the mandibular molar tooth extraction forceps were soaked in ozonized water for 30 minutes. ^15^ In the 4.8% chloroxylenol treatment group, mandibular molar extraction forceps were soaked for 60 minutes in 250 mL 4.8% chloroxylenol. ^7^ In the negative control group (Aquadest) the forceps were rinsed with distilled water without using soap or antiseptic solution at all. After disinfection, the forceps were removed from the disinfection container using tool tongs and dried with sterile gauze. The mandibular molar tooth extraction forcep is treating individually.

### Taking the samples

Sampling was carried out by immersing mandibular molar extraction forceps in a container containing 50 mL of saline solution for five minutes, then covered with aluminum foil. The forceps are removed from the sample container and the container is closed again with plastic wrap and labeled for each sample. Group label for ozonized water was Z, 4.8% chloroxylenol was K, and distilled water was A. ^10^

### Counting bacterial colonies

The samples were then taken to the Faculty of Pharmacy, Mathematics and Natural Science, University of Sumatera Utara, to count the number of bacterial colonies. Samples were diluted using NaCl as much as 10 ^3^, then implanted in a petri dish containing plant count agar (PCA) media, using the spread plate method. A spread method plate is a technique for growing microorganisms in agar media isolate or count the bacteria present. ^14^ The sample was spread using a sterile spreader, and the petri dish was rotated at an angle of 45° above a Bunsen burner. After that, the petri dishes were incubated in the incubator using an autoclave at a temperature of 37°C for 24 hours. Following this, the number of bacterial colonies was counted using a bacterial colony counter.

### Statistical analysis

**Figure 1.**
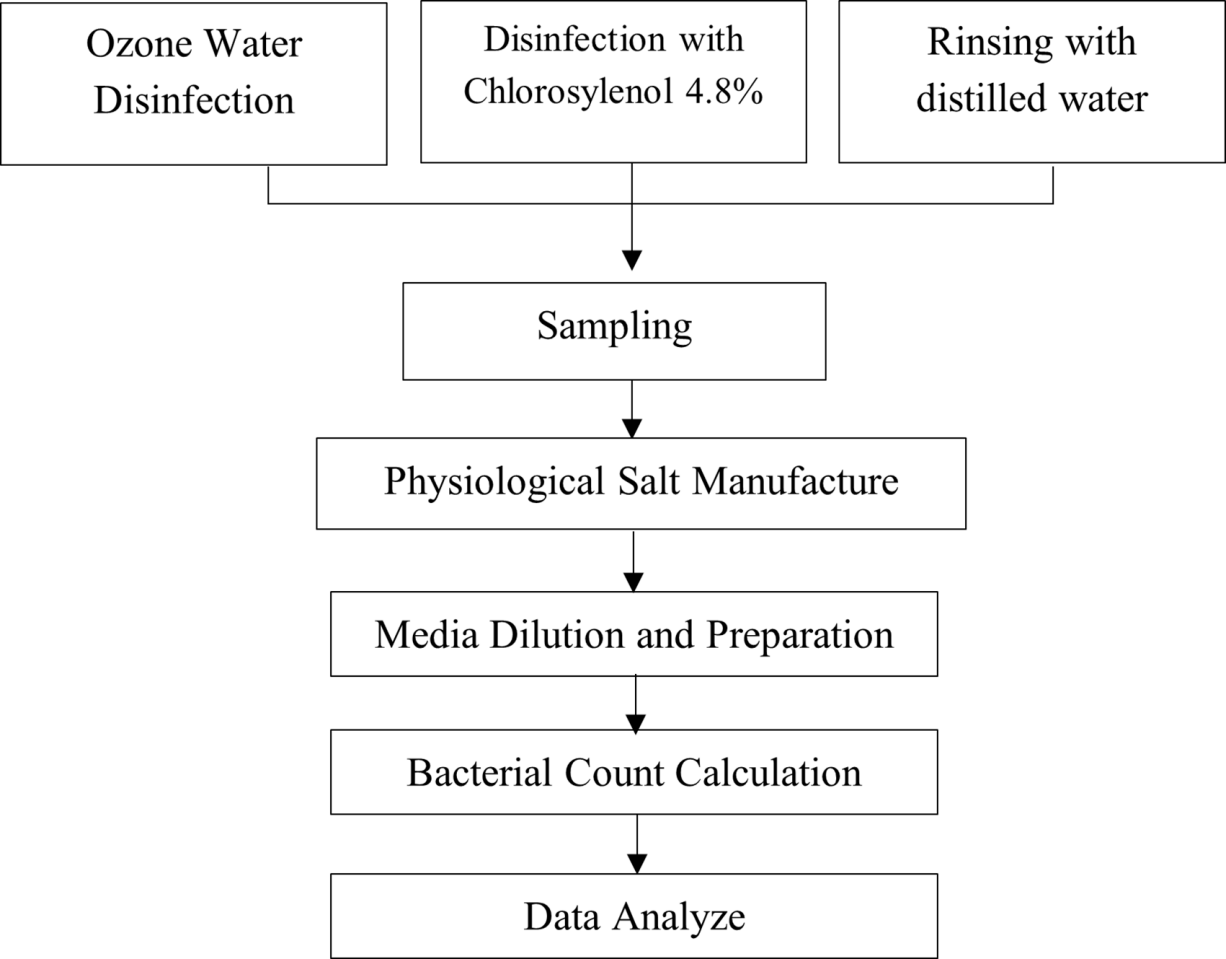
Research design.

Data analysis was performed using the SPSS version 22.0 software. The normality of the data was tested using the Shapiro-Wilk test, ^9^ since the number of samples was less than 50. The results of the data normality test showed that the data were not normally distributed, with p-values with a degree of significance < 0.05; therefore the next test used was the non-parametric Kruskal Wallis test followed up by the Mann-Whitney U test with a significance degree of p ≤ 0.05, to find out which treatment group had the best effectiveness as a disinfecting agent ( Figure 1). ^16^

## Results

This study consisted of 30 mandibular molar extraction forceps with three treatment groups, namely the mandibular molar extraction forceps group which was disinfected with ozonized water (Z), the mandibular molar forceps group which was disinfected with 4.8% chloroxylenol solution (K), and the mandibular molar forceps group which was rinsed with distilled water (A) as a negative control. ^17^ Each group consisted of 10 mandibular molar extraction forceps. ^18^

The 10 mandibular molar-removing forceps that were disinfected with ozonized water had a mean bacterial count of 4.00 ± 4.32 CFU/mL, showing they were still contaminated with pathogenic microorganisms with the highest number of bacterial colonies being 11·10 ^3^ CFU/mL. ^19^ The 10 forceps which were disinfected with 4.8% chloroxylenol had a mean bacterial count of 16.00 ± 6.65 CFU/mL, showing they were still contaminated with pathogenic microorganisms, with the highest number of bacterial colonies being 27·10 ^3^ CFU/mL. ^20^ Meanwhile, the mean bacterial count in the negative control group (Aquades) the 10 forceps extracting mandibular molars showed 217.50 ± 39.24 CFU/mL still forming bacterial colonies, with the highest number of bacterial colonies being 292·10 ^3^ CFU/mL ( Table 1). ^14^

**Table 1.** Number of bacterial colonies (10 ^3^ × CFU/mL).

Code	Ozone water	4.8% Chloroxylenol	Aquadest
1	0	14	292
2	0	15	230
3	0	5	257
4	4	11	236
5	0	13	195
6	5	22	253
7	11	12	270
8	9	27	191
9	2	24	205
10	9	17	173

Based on the Kruskal Wallis statistical test results the effectiveness of ozone water, 4.8% chloroxylenol and negative control (Aquadest) on the number of bacterial colonies in mandibular tooth extraction forceps was significantly different, with a significance value of p = 0.000 (p < 0.05) ( Table 2). ^4^

**Table 2.** Kruskal Wallis test results for ozone water, 4.8% chloroxylenol, and negative control (Aquadest).

Treatment	Mean ± SD	p-value
Ozone water	4.00 ± 4.32	0.000
4.8% Chloroxylenol	16.00 ± 6.65	
Negative control (Aquadest)	217.50 ± 39.24	

Based on the results of a further using the Mann-Whitney U test comparing disinfection effectiveness between groups treated with ozone water disinfection and negative control (Aquadest), the significance value was p = 0.000 (p < 0.05). ^21^ Ozone water was more effective as a disinfection agent compared to negative control on the number of bacterial colonies in dental extraction instruments at the Universitas Sumatera Utara Dental and Oral Hospital, as indicated by the lower average value of bacterial counts in the ozone water group, which was 4.00 ± 4.32 CFU/mL. In contrast, for the negative control group (Aquadest) the value was 217.50 ± 39.24 ( Table 3).

**Table 3.** Test results of the Mann-Whitney U comparison of ozone water and negative control (Aquadest).

Treatment	Mean	p-value
Ozone water	4.00 ± 4.32	0.000
Negative control (Aquadest)	217.50 ± 39.24	

 Further Mann-Whitney U test results showed that there was a significant difference between the effectiveness of disinfection between groups treated with 4.8% chloroxylenol and negative control with a significance value of p = 0.000 (p < 0.05). 4.8% chloroxylenol was more effective as a disinfecting agent compared to negative control on the number of bacterial colonies in dental extraction instruments. ^22^ The Universitas Sumatera Utara indicated that the average bacterial count value of the 4.8% chloroxylenol group was lower with 16 ± 6.65 CFU/mL, while the value for the negative control group was 217.50 ± 39.24 ( Table 4).

**Table 4.** Test results of the Mann-Whitney U comparison of 4.8% chloroxylenol, and negative control (Aquadest).

Treatment	Mean ± SD	p-value
4.8% Chloroxylenol	16.00 ± 6.65	0.000
Negative control (Aquadest)	217.50 ± 39.24	

Further Mann-Whitney U test results showed that there was a significant difference in effectiveness between ozone water and 4.8% chloroxylenol with a significance value of p = 0.000 (p < 0.05). ^23^ Ozone water was more effective as a disinfection agent compared to 4.8% chloroxylenol against bacterial colonies on dental extraction instruments, as indicated by the lower average bacterial count value of the ozone water group which was 4.00 ± 4.32 CFU/mL, while for the disinfection treatment group with 4.8% chloroxylenol, the value was 16 ± 6.65 CFU/mL ( Table 5). ^24^

**Table 5.** Mann-Whitney U test results comparing ozone water and chloroxylenol 4.8%.

Treatment	Mean	p-value
Ozone water	4.00 ± 4.32	0.000
4.8% chloroxylenol	16.00 ± 6.65	

**Figure 2.**
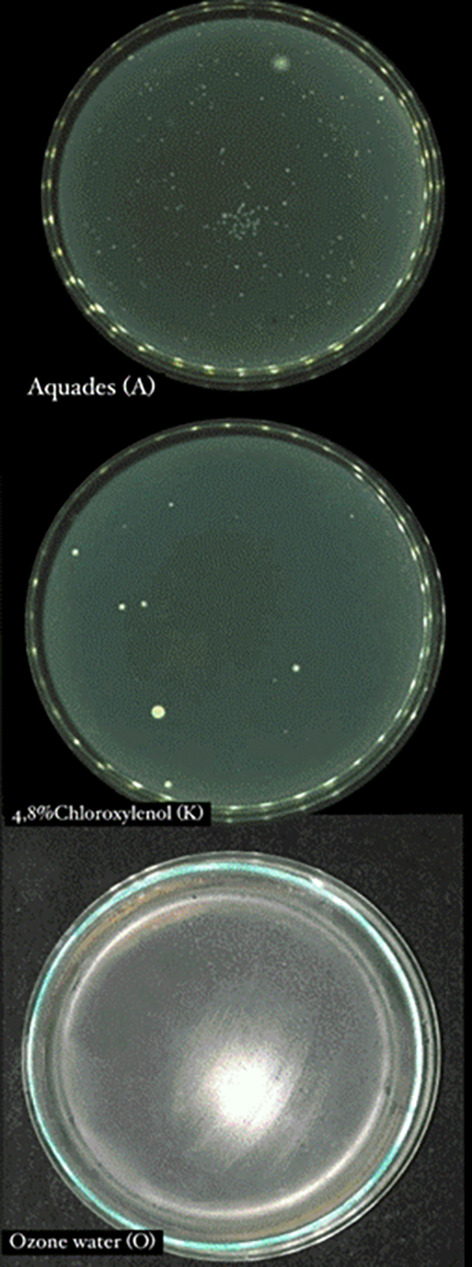
Total plate count 4.8% chloroxylenol, aquadest, and ozone water.

Based on the results of data analysis from each treatment group, it was shown that ozone water was significantly more effective in disinfecting mandibular molar tooth extraction forceps compared to chloroxylenol 4.8% and negative control. ^25^ Instruments in the 4.8% chloroxylenol group showed a smaller number of bacterial colonies than the negative control ( Figure 2).

## Discussion

This study compared two disinfection solutions, namely ozone water and 4.8% chloroxylenol. Ozone water is a strong alternative to disinfectants and effectively kills pathogenic microorganisms such as bacteria, viruses, fungi, protozoa, and endospores. ^6^ Ozone is more effective against large numbers of microorganisms because of its ability to oxidize microorganisms without causing resistance. Ozone can inhibit the control of enzymes and therefore act on bacterial cell metabolism and damage bacterial cell membranes. Ozone water disrupts the integrity of the bacterial cell envelope through the oxidation of phospholipids and lipoproteins.

In this study, the concentration of ozone water used was 15 mg/L, with a soaking time of 30 minutes. The use of ozone water as a material for disinfecting dental instruments was recommended at a concentration of 10-20 mg/L. As reported by previous research, a concentration of ozone water that is too low showed a reduction in the antibacterial effectiveness of ozone water, while a concentration that is too high and exposure for too long could cause toxicity. Ozone maintained its antibacterial properties for the first 20 minutes, and after 30 minutes the stability of ozone water slowly decreased. After eight hours, there was no more ozone in the ozonized water.

Based on the data on the number of bacterial colonies in the treatment group with ozone water samples, ozone water was more effective in disinfecting the mandibular molar forceps. It was observed that four out of 10 mandibular molar extraction forceps were successfully decontaminated, with six samples showing bacterial growth with the highest number of bacteria being 11·10 ^3^ CFU/mL. The number of bacterial colonies in the treatment group with ozone water was the lowest when compared to the other two treatment groups, with an average value of 4.00 ± 4.32. This is due to the greater efficiency of ozone water in killing bacterial endospores by oxidizing and damaging the bacterial membrane which contains lipoproteins and fatty acids, so that the bacteria experience failure in the germination process.

In contrast, 4.8% chloroxylenol is unable to kill bacterial endospores, therefore 4.8% chloroxylenol is still classified as medium-level chemical disinfection. This makes ozone water more effective in killing microorganisms when compared to 4.8% chloroxylenol according to César *et al.*, 2012, who stated that the antimicrobial activity of ozone water against *B. atrophaeus* spores resulted in a decrease in growth of 90.15% and 98.74% after 10 and 30 minutes of exposure to ozone water, respectively. In this study, the average number of bacterial colonies in the ozone water treatment group was lower due to the ability of the ozone water to kill bacterial endospores. Endospora is very resistant to high temperatures, extreme environmental conditions, or chemicals. The ability of ozone water to kill bacterial endospores was likely one of the causes of the fewer bacterial colonies formed in the ozone water treatment group. Data from César *et al.* also shows that the use of ozonated water (10 mg/L) for 30 minutes was effective for disinfection of diamond burs that have been contaminated by *S. aureus*, *E. coli*, *C. albicans*, and spores of *B. atrophaeus*, reducing bacterial contamination by 90.15-99.33%. In addition, based on Bezirtzoglou *et al.* found the research of César *et al.* ^10^ states that a few teeth that have been soaked for 30 minutes with ozone water showed no bacterial colonies formed on the toothbrush. In the research by Nielsen *et al.*, 2007, the addition of ozone to water that has been inoculated with *S. aureus* showed a reduction of 98.9%; *S. faecalis*, *P. aeruginosa* were respectively reduced by 64.2% and 57.4%. ^26^ According to Xiao Hu *et al.*, 2021, ozone water can eliminate severe acute respiratory syndrome coronavirus 2 (SARS-CoV-2) with an ozone concentration above 18 mg/L within one minute. ^15^ Wood *et al.* stated that ozone water can reduce fungal spores by 50.7-91.2%. Thus, ozone water can kill microorganisms in the form of bacteria, viruses, fungi, and bacterial endospores. ^27^

In the 4.8% chloroxylenol treatment group, there was bacterial growth in all mandibular molar tooth extraction forceps with a lower value compared to ozone water, but the number of bacterial colonies formed was less than the negative control. The mean bacterial count value in the 4.8% chloroxylenol group was 16.00 ± 6.65, while for the negative control group, the value was 217.50 ± 39.24. The research by Xiang *et al.* in 2018 showed that 11 out of 18 mandibular molar tooth extraction forceps which were cleaned using 4.8% chloroxylenol did not form bacterial colonies, and seven mandibular molar tooth extraction forceps had a maximum bacterial count of 812·10 ^3^ CFU/mL and the average number of bacterial colonies formed was 82.5. This is by the results of this study: in all research samples bacterial colonies still formed although with fewer bacterial colonies on average. ^28^ Liu *et al.*, 2020, who researched the skin and the hospital environment, found that 4.8% chloroxylenol could interfere with the metabolic activity of Gram-positive bacteria by 38.9% and Gram-negative by 60.7%. ^29^ Mohammed AL-jaleel Khalil *et al.*, 2023, showed that 4.8% chloroxylenol was effective in killing 100% isolates of *Staphylococcus* sp. and *Streptococcus* sp. Thus, 4.8% chloroxylenol was able to kill pathogenic microorganisms. Still, its effectiveness is lower when compared to ozone water. The -OH hydroxyl group of the 4.8% chloroxylenol molecule binds to proteins in the bacterial cell membrane to disrupt it, thereby allowing the contents of the bacterial cell to leak. This allows 4.8% chloroxylenol to enter the bacterial cell to bind more proteins and enzymes and deactivate cell functions. Meanwhile, the negative control group (Aquadest) had a lethal effect on microorganisms because distilled water is a neutral organic compound used as a pure solvent and does not have antibacterial activity. ^20^

Differences in the number of bacterial colonies can also be caused by several factors, one of which is the number of microorganisms and contaminants, the condition of the patient’s oral cavity, the number and type of normal flora in the oral cavity of each patient which can differ due to severe caries, periodontal disease, pulpal necrosis or abscess at the time of tooth extraction. ^4^ Disinfectants have different abilities and susceptibility depending on the number and type of bacteria present in the patient’s oral cavity; the duration of exposure and the concentration of the disinfecting agents also affect the ability to kill microorganisms. ^8^ The decrease in the number of different bacterial colonies in each treatment is also influenced by open environmental conditions, environmental temperature, and humidity both during sampling and when processing samples in the laboratory, ozone generator output, and operator negligence.

## Conclusions

Ozone water was significantly more effective in disinfecting dental extraction instruments at Universitas Sumatera Utara Dental and Oral Hospital than 4.8% chloroxylenol with a significance value of p = 0.000 (p < 0.05).

## Data availability

### Underlying data

Zenodo: The Data Set “Ozone Water And 4.8% Chloroxylenol Against The Number Of Bacterial Colonies In Dental Extraction Instruments at The Universitas Sumatera Utara Dental and Oral Hospital”, https://doi.org/10.5281/zenodo.7950593. ^30^

This project contains the following underlying data:

• Raw data of total plate count aquades.csv (Raw data of total plate count aquades)
• Raw data of total plate count chloroxylenol- Sheet1.csv (Raw data of total plate count 4.8% choloroxylenol)
• Raw data of total plate count.ozone water.csv (Raw data of total plate count.ozone water)
• Result of Normalitas Test.csv (raw data of result of normalitas test with shapiro wilk)
• Result of Kruskal wallis test.csv (raw data of result of kruskal wallis)
• Result of comparison ozone water and Choloroxylenol.csv (raw data of result of comparison ozon water and 4,8% chloroxylenol)
• Result of Comparison Choloxylenol and Aquades.csv (raw data of result of comparison 4,8% chloroxylenol and aquades)
• Result of Comparison ozone water and Aquades.csv (raw data of result of comparison ozone water and aquades)
• Z1-10.rar (image of colony bactery plate of ozone water)
• Choloroxylenol.jpg (image of colony bactery plate of 4,8% chloroxylenol)
• Aquades.jpg (image of colony bactery plate of aaquades)

Data are available under the terms of the Creative Commons Attribution 4.0 International license (CC-BY 4.0).
