# Supplementary material for: Ozone water or chloroxylenol: The comparison of disinfection effectiveness against the number of bacterial colonies in dental extraction instruments at the Universitas Sumatera Utara Dental and Oral Hospital
Source: F1000Res. 2025 Jul 31;12:726. Originally published 2023 Jun 21. [Version 4] doi: 10.12688/f1000research.132941.4 (PMC12374152; doi:10.12688/f1000research.132941.4)
Supplement: Peer review history

# Reviewer report: Reviewer response for version 4

**DOI:** 10.5256/f1000research.182380.r405353

**Published:** 2025-08-23

**Authors:** Dario Gaudioso

**Affiliations:** 1 Università degli Studi di Firenze, Sesto Fiorentino, Firenze, Italy

Paper's experimental simplicity is very clear. It compares the use of ozonated water with the traditional treatment with chloroxylenol 4.8%. There are some points that would have been interesting to explore further, such as a more thorough screening of the genera of microorganisms that survive the different treatments. In parts of the paper, the genus and species are not italicized. Methodologically, some literature reports that the effect of ozone after 5 minutes is not effective, contrary to what is reported here. Overall, the paper, despite its operational and experimental simplicity, presents some interesting key points.

Is the work clearly and accurately presented and does it cite the current literature?

Yes

If applicable, is the statistical analysis and its interpretation appropriate?

Yes

Are all the source data underlying the results available to ensure full reproducibility?

Yes

Is the study design appropriate and is the work technically sound?

Yes

Are the conclusions drawn adequately supported by the results?

Yes

Are sufficient details of methods and analysis provided to allow replication by others?

Yes

Reviewer Expertise:

Molecular biology and microbiology

I confirm that I have read this submission and believe that I have an appropriate level of expertise to confirm that it is of an acceptable scientific standard.

# Reviewer report: Reviewer response for version 2

**DOI:** 10.5256/f1000research.158749.r319597

**Published:** 2024-09-20

**Authors:** Beni Jequicene Mussengue Chaúque

**Affiliations:** 1 Universidade Rovuma, Niassa Branch, Lichinga, Mozambique; 2 Universidade Federal do Rio Grande do Sul, Porto Alegre, Brazil

The MS titled “Ozone Water or Chloroxylenol: A Comparison of Disinfection Effectiveness Against Bacterial Colonies on Dental Extraction Instruments at the USU Dental and Oral Hospital” explores the effectiveness of ozone water and chloroxylenol in inactivating bacteria on dental instruments.

**General comments**

 While the topic is of considerable interest, the study suffers from several key issues, particularly in the writing and methodology sections, which are presented in an unstructured way. Moreover, the study's overall contribution to the existing literature appears limited.

 The sampling procedures are unclear, and the authors do not provide sufficient detail on how they minimized the risk of bias in the allocation of instrument samples. With only 10 samples, the study’s findings are not robust enough to justify any broad generalizations.

 Another major limitation is the lack of identification and/or isolation of microorganisms, which would have allowed for a more standardized evaluation of disinfectant performance against comparable inoculum sizes. Additionally, the washing processes used prior to instrument handling, to remove blood and saliva, could introduce bias especially given the small sample size.

**Specific comments**

**Title**

 1- It is not advisable to use abbreviations in the title. I suggest either writing "USU" in full or omitting it entirely.

**Abstract**

 2 -The rationale for conducting the study is not clearly established. Please elaborate further. For example, isn't the use of ozone water and 4.8% chloroxylenol already a proven and standardized aseptic practice? If so, why is this study necessary?

 3 -Additionally, the abbreviation "USU" appears in the abstract without being explained.

 4 -“This study aimed to evaluate the effectiveness of ozone water and 4.8% chloroxylenol in reducing bacterial colonies on dental extraction instruments at the USU Dental and Oral Hospital between October and December 2022.” Note: The phrase "between October and December 2022" seems unnecessary here and can be removed.

 5 -The methods section of the abstract should be written more formally, detailing the sampling, transportation, treatment (including the form, application, and exposure), and the plate inoculation process.

 6 -The study is based on only 10 different instruments. Why such a small sample size? How representative is this sample?

 7 -The results are reported in a confusing manner. The p-value analysis is presented before the colony count values. The authors present two values for the number of colonies in the treatment group: 4.00 ± 4.32 and 16.00 ± 6.65. Which value is correct? If the values refer to ozone water and chloroxylenol, respectively, please specify this clearly.

**Introduction**

 8 -Paragraphs 1, 2, and 3 contain redundant information. Please consider rewriting to avoid repetition.

 9 - Note: Only separate paragraphs when necessary for clarity.

 10 - In paragraph 5, ensure the names of microorganisms are spelled correctly.

**Methods**

 11 - The sentence "The patient’s oral cavity state, the kind and quantity of bacteria present, the surrounding circumstances, and the instrument settings before tooth extraction are all non-parametric" is unclear. This sounds more like a result rather than a method. Please consider rewriting

 12 - The following information needs to be placed in the appropriate section: "The study parameters include the concentration of ozone water (15 mg/L), the concentration of chloroxylenol (4.8%), the immersion time in ozone water (30 minutes), the immersion time in chloroxylenol solution (60 minutes), and the pre-treatment procedures." This information is also repeated in the "Disinfection procedure" section.

 13 - Instead of simply stating that " The sampling technique used in this study was purposive sampling" provide a detailed, step-by-step explanation of the sampling process to ensure reproducibility by other researchers.

 14 - The subsections “Disinfection procedure” and “Taking the samples” are unclear and need to be rewritten in a more straightforward, didactic manner. Clearly distinguish between the different types of samples used in the study, such as instruments and inoculum. Include specific details regarding their identification, selection, and collection.

 15 - It is also unclear whether the instruments were treated individually or as a group.

 16 - In the “Counting bacterial colonies” subsection, specify the volume of the sample plated and the incubation temperature.

**Results**

 17 - The results in Tables 2–5 would be easier to understand if presented in a graph, allowing readers to visualize and compare the data more effectively.

**Conclusion**

 18 - The statement “Ozone water can kill bacterial endospores while 4.8% chloroxylenol cannot” should not be included in the conclusions, as this was not assessed in the study.

Is the work clearly and accurately presented and does it cite the current literature?

Partly

If applicable, is the statistical analysis and its interpretation appropriate?

Yes

Are all the source data underlying the results available to ensure full reproducibility?

No

Is the study design appropriate and is the work technically sound?

Partly

Are the conclusions drawn adequately supported by the results?

Partly

Are sufficient details of methods and analysis provided to allow replication by others?

Partly

Reviewer Expertise:

Microbiology and Pharmacology

I confirm that I have read this submission and believe that I have an appropriate level of expertise to state that I do not consider it to be of an acceptable scientific standard, for reasons outlined above.

# Response

**Published:** 2025-04-03

**Authors:** Hendry Rusdy

**Affiliations:** Department of Oral and Maxillo, Universitas Sumatera Utara, Medan, North Sumatra, Indonesia

**General comments**

 The amount of sample is 30 

**Specific comments**

**Title**

 Change USU to University of South Sumatera

**Abstract**

 2 -The rationale for conducting the study is not clearly established. Please elaborate further. For example, isn't the use of ozone water and 4.8% chloroxylenol already a proven and standardized aseptic practice? If so, why is this study necessary? **answer** :  Research on ozone water as a disinfectant is still limited and no research has been found on the use of ozone water on tooth extraction instruments. However, ozone has antibacterial properties so researchers are interested in studying the effectiveness of ozone water as a disinfectant when compared to the commonly used 4.8% chloroxylenol

 3 - "USU" appears in the abstract without being explained.

 4 -The phrase "between October and December 2022" seems unnecessary here and can be removed.

 5 -The methods has been updated

 6 -The study is based on only 10 different instruments. Why such a small sample size? How representative is this sample? **answer : The amount of sample is 30**

 7 -The results are reported in a confusing manner. The p-value analysis is presented before the colony count values. The authors present two values for the number of colonies in the treatment group: 4.00 ± 4.32 and 16.00 ± 6.65. Which value is correct? If the values refer to ozone water and chloroxylenol, respectively, please specify this clearly. **answer** : value refer to ozone water, which is 4.00 ± 4.32

**Introduction**

 8 -Paragraphs 1, 2, and 3 has been updated

 9 - updated

 10 - In paragraph 5, ensure the names of microorganisms are spelled correctly has been updated

**Methods**

 11 - The sentence "The patient’s oral cavity state, the kind and quantity of bacteria present, the surrounding circumstances, and the instrument settings before tooth extraction are all non-parametric" is unclear. This sounds more like a result rather than a method. Please consider rewriting  **have been updated**

 12 - The following information needs to be placed in the appropriate section: "The study parameters include the concentration of ozone water (15 mg/L), the concentration of chloroxylenol (4.8%), the immersion time in ozone water (30 minutes), the immersion time in chloroxylenol solution (60 minutes), and the pre-treatment procedures." This information is also repeated in the "Disinfection procedure" section  **have been updated**

 13 - Instead of simply stating that " The sampling technique used in this study was purposive sampling" provide a detailed, step-by-step explanation of the sampling process to ensure reproducibility by other researchers. **have been updated**

 14 - The subsections “Disinfection procedure” and “Taking the samples” are unclear and need to be rewritten in a more straightforward, didactic manner. Clearly distinguish between the different types of samples used in the study, such as instruments and inoculum. Include specific details regarding their identification, selection, and collection.  **have been updated**

 15 - It is also unclear whether the instruments were treated individually or as a group.  **have been updated**

 16 - In the “Counting bacterial colonies” subsection, specify the volume of the sample plated and the incubation temperature.  **have been updated**

**Results**

 17 - The results in Tables 2–5 would be easier to understand if presented in a graph, allowing readers to visualize and compare the data more effectively.  **have been updated**

**Conclusion**

 18 - The statement “Ozone water can kill bacterial endospores while 4.8% chloroxylenol cannot” should not be included in the conclusions, as this was not assessed in the study.  **have been updated**

# Reviewer report: Reviewer response for version 2

**DOI:** 10.5256/f1000research.158749.r282421

**Published:** 2024-06-06

**Authors:** Shorouq Abass

**Affiliations:** 1 Department of Prosthodontics, College of Dentistry, University of Baghdad, Baghdad, Baghdad Governorate, Iraq

Dear Authors, thank you for this interesting article. Here are some of the comments of mine:

 In the introduction section you have to mention other aspect of disinfection solution, technique, and dental materials in general other than of your specific subject of research. e.g.  

- Khalaf BS, Abass SM, Al-Khafaji AM, and Issa MI, Antimicrobial efficiency of hypochlorous acid and its effect on some properties of alginate impression material. Int J Dent, 2023. 2023: p. 1-7 DOI: 10.1155/2023/8584875.
- Al-khafaji AM, Abass SM, and Khalaf BS, The effect of SOLO and sodium hypochlorite disinfectant on some properties of different types of dental stone. J Bagh Coll Dent, 2013. 25(2): p. 8-17
- Mahmood MA, Khalaf BS, and Abass SM, Efficiency of different denture disinfection methods. Global J Bio-sci Biotech, 2017. 6: p. 439-4.
  It is only a suggestion for authors to use the above citations because they are related to the subject of disinfection in dentistry in general and the authors can refer to it if they wish. 

Is the work clearly and accurately presented and does it cite the current literature?

Partly

If applicable, is the statistical analysis and its interpretation appropriate?

Partly

Are all the source data underlying the results available to ensure full reproducibility?

Partly

Is the study design appropriate and is the work technically sound?

Partly

Are the conclusions drawn adequately supported by the results?

Yes

Are sufficient details of methods and analysis provided to allow replication by others?

No

Reviewer Expertise:

Prosthodontics, dental materials, disinfection and dental implant.

I confirm that I have read this submission and believe that I have an appropriate level of expertise to confirm that it is of an acceptable scientific standard, however I have significant reservations, as outlined above.

## References

[1] : Antimicrobial Efficiency of Hypochlorous Acid and Its Effect on Some Properties of Alginate Impression Material. *Int J Dent* .2023;2023: 10.1155/2023/8584875 8584875 10.1155/2023/8584875 38021346 PMC10663091

[2] : The effect of SOLO and sodium hypochlorite disinfectant on some properties of different types of dental stone. *University of Baghdad* .1973;8-17

[3] : Efficiency of different denture disinfection methods. *Global J Bio-sci Biotech* .2017;439-439

# Reviewer report: Reviewer response for version 1

**DOI:** 10.5256/f1000research.145897.r207744

**Published:** 2023-10-09

**Authors:** Paulo J. Palma

**Affiliations:** 1 Institute of Endodontics, Faculty of Medicine, University of Coimbra, Coimbra, Portugal

The present study aimed to determine the effectiveness of disinfection of ozone water and 4.8% chloroxylenol  in reducing the number of bacterial colonies on dental extraction instruments at the USU Dental and Oral Hospital between October-December 2022..

 This research is under the scope of this journal; the topic is relevant for readers, and this research deals with potentially significant knowledge of the field. And It will be important for knowledge. The topic is relevant for readers and this review deals with potentially significant knowledge in the field and opens new ways for future studies.

 However, there are some aspects that are possibly improved in the manuscript:

**Keywords**

- Please add more keywords, and order the keywords / Mesh terms alphabetically

**Introduction**

- What is the importance of this study? Which results are comparable with other articles? What has this study been new?

**M&M**

- **​​​**A flowchart of all experimental study would be valuable.
- How many slides per sample obtained, and how many of them were evaluated? How were the sample cells calculated? Did authors performed power analysis to evaluate if this sample size was appropriate?

**Analysis:**

- How many observers did the scoring? What is the interobserver agreement? How many time was performed done the determinations?
- Which data are parametric and non-parametric? Please specify?
- When mentioning materials or devices: for some of them you don't mention the manufacturer at all, for some you mention only the manufacturer, for some the manufacturer and city, for some you mention the manufacturer and city/ country.

**Discussion**

-  Please, clarified more limitations of this study? 
- An objective presentation of the strengths and limitations of the model, study design and methods, must be provided, including any biology/functional variability between the animal model and humans.
- And also clarified the future perspectives in the discussion. The potential influence of the results on future research plans needs also be discussed.

Is the work clearly and accurately presented and does it cite the current literature?

Partly

If applicable, is the statistical analysis and its interpretation appropriate?

Partly

Are all the source data underlying the results available to ensure full reproducibility?

Partly

Is the study design appropriate and is the work technically sound?

Partly

Are the conclusions drawn adequately supported by the results?

Partly

Are sufficient details of methods and analysis provided to allow replication by others?

Partly

Reviewer Expertise:

Dentistry, infection control

I confirm that I have read this submission and believe that I have an appropriate level of expertise to confirm that it is of an acceptable scientific standard, however I have significant reservations, as outlined above.

# Reviewer report: Reviewer response for version 1

**DOI:** 10.5256/f1000research.145897.r193467

**Published:** 2023-08-08

**Authors:** Shorouq Abass

**Affiliations:** 1 Department of Prosthodontics, College of Dentistry, University of Baghdad, Baghdad, Baghdad Governorate, Iraq

The research needs careful attention to the following:

1. Please reduce the words number of the research title “ The disinfection effectiveness of ozone water and  4.8% chloroxylenol against the number of bacterial colonies in dental extraction instruments at the USU Dental and  Oral Hospital in October-December 2022” in to at least 12 words number.
2. In introduction you stated that “This study aimed to compare the effectiveness of **ozone water** and 4.8% chloroxylenol disinfectants on tooth extraction instruments, because these critical instruments have a high risk of causing infection as they penetrate the mucous membranes of the oral cavity” As shown in the bolded text you didn’t specify the concentration of the ozone in the water, so please correct it.
3. In method and in subheading “Sampling” you stated that “The sampling technique used in this study was purposive sampling with **30 mandibular molar tooth extraction pliers** as the sample….” As shown in the bolded text you didn’t specify the sample collection technique, please correct it.
4. In method and in subheading “Ozone water production” you stated that “Ozone water was prepared using an **ozone generator** for 20 minutes with an ozone water concentration of **15 mg/L**” as shown in the bolded text you didn’t specify the ozone generation method, please correct it.
5. In method and in the subheading  “Disinfection procedure” you stated that “In the ozone water and 4.8% chloroxylenol treatment group, the mandibular molar tooth extraction pliers were **rinsed under running water, brushed with an antiseptic solution and rinsed again until the pliers were free of blood and saliva before soaking in a disinfecting solution…**” as shown in the bolded text you didn’t specify the time and the duration of rinse with water, method of brush, type of antiseptic, please correct it. Also you stated that “After that, the mandibular molar tooth extraction forceps were soaked in ozonized water for 30 minutes and then a **sample was taken** ..” how you take the sample , please correct it with details.
6. In method and in subheading “Counting bacterial colonies” you stated “……… a **bacterial colony counter**” as shown in the yellow highlight you didn’t specify the type of the counter and the type of bacteria or otherwise your test is not specified to specific type of bacteria and you only have a negative or positive growth.
7. In the result section your SD was too high more than 2, please can you state why?

Is the work clearly and accurately presented and does it cite the current literature?

Partly

If applicable, is the statistical analysis and its interpretation appropriate?

Partly

Are all the source data underlying the results available to ensure full reproducibility?

Partly

Is the study design appropriate and is the work technically sound?

Partly

Are the conclusions drawn adequately supported by the results?

Yes

Are sufficient details of methods and analysis provided to allow replication by others?

No

Reviewer Expertise:

Prosthodontics, dental materials, disinfection and dental implant.

I confirm that I have read this submission and believe that I have an appropriate level of expertise to confirm that it is of an acceptable scientific standard, however I have significant reservations, as outlined above.
