# Supplementary material for: Ozone water or chloroxylenol: The comparison of disinfection effectiveness against the number of bacterial colonies in dental extraction instruments at the Universitu of South Sumatera Dental and Oral Hospital
Source: F1000Res. 2025 May 12;12:726. Originally published 2023 Jun 21. [Version 3] doi: 10.12688/f1000research.132941.3 (PMC12374152; doi:10.12688/f1000research.132941.3)
Supplement: Peer review history

# Response

**Published:** 2025-04-03

**Authors:** Hendry Rusdy

**Affiliations:** Department of Oral and Maxillo, Universitas Sumatera Utara, Medan, North Sumatra, Indonesia

**General comments**

 The amount of sample is 30 

**Specific comments**

**Title**

 Change USU to University of South Sumatera

**Abstract**

 2 -The rationale for conducting the study is not clearly established. Please elaborate further. For example, isn't the use of ozone water and 4.8% chloroxylenol already a proven and standardized aseptic practice? If so, why is this study necessary? **answer** :  Research on ozone water as a disinfectant is still limited and no research has been found on the use of ozone water on tooth extraction instruments. However, ozone has antibacterial properties so researchers are interested in studying the effectiveness of ozone water as a disinfectant when compared to the commonly used 4.8% chloroxylenol

 3 - "USU" appears in the abstract without being explained.

 4 -The phrase "between October and December 2022" seems unnecessary here and can be removed.

 5 -The methods has been updated

 6 -The study is based on only 10 different instruments. Why such a small sample size? How representative is this sample? **answer : The amount of sample is 30**

 7 -The results are reported in a confusing manner. The p-value analysis is presented before the colony count values. The authors present two values for the number of colonies in the treatment group: 4.00 ± 4.32 and 16.00 ± 6.65. Which value is correct? If the values refer to ozone water and chloroxylenol, respectively, please specify this clearly. **answer** : value refer to ozone water, which is 4.00 ± 4.32

**Introduction**

 8 -Paragraphs 1, 2, and 3 has been updated

 9 - updated

 10 - In paragraph 5, ensure the names of microorganisms are spelled correctly has been updated

**Methods**

 11 - The sentence "The patient’s oral cavity state, the kind and quantity of bacteria present, the surrounding circumstances, and the instrument settings before tooth extraction are all non-parametric" is unclear. This sounds more like a result rather than a method. Please consider rewriting **have been updated**

 12 - The following information needs to be placed in the appropriate section: "The study parameters include the concentration of ozone water (15 mg/L), the concentration of chloroxylenol (4.8%), the immersion time in ozone water (30 minutes), the immersion time in chloroxylenol solution (60 minutes), and the pre-treatment procedures." This information is also repeated in the "Disinfection procedure" section **have been updated**

 13 - Instead of simply stating that " The sampling technique used in this study was purposive sampling" provide a detailed, step-by-step explanation of the sampling process to ensure reproducibility by other researchers. **have been updated**

 14 - The subsections “Disinfection procedure” and “Taking the samples” are unclear and need to be rewritten in a more straightforward, didactic manner. Clearly distinguish between the different types of samples used in the study, such as instruments and inoculum. Include specific details regarding their identification, selection, and collection. **have been updated**

 15 - It is also unclear whether the instruments were treated individually or as a group. **have been updated**

 16 - In the “Counting bacterial colonies” subsection, specify the volume of the sample plated and the incubation temperature. **have been updated**

**Results**

 17 - The results in Tables 2–5 would be easier to understand if presented in a graph, allowing readers to visualize and compare the data more effectively. **have been updated**

**Conclusion**

 18 - The statement “Ozone water can kill bacterial endospores while 4.8% chloroxylenol cannot” should not be included in the conclusions, as this was not assessed in the study. **have been updated**
